# Editorial

**Published:** 1896-06

**Authors:** 


### HOMŒOPATHIC PHYSICIAN,

A MONTHLY JOURNAL OF

HOMŒOPATHIC MATERIA MEDICA AND CLINICAL MEDICINE.

"If our school ever gives up the strict inductive method of Hahnemann, we are lost, and deserve only to be mentioned as a caricature in the history of medicine."—CONSTANTINE HERING.

Vol. XVI. JUNE, 1896. No. 6.

### EDITORIALS.

MERCURIUS-VIRUS.—Mercurius is often indicated in inflammation of the ear, with discharge of pus from the ear, and external ulceration. Chamomilla is to be thought of when the pus is thin. Silicea when the pus is watery and offensive. Pulsatilla when the pus is thick and green or greenish-yellow. Calcarea-carbonica for white pus with profuse perspiration about the head, having a sour smell, coming on when the child is sleeping.

The editor made a brilliant cure of a case of this kind in a little child by using Calcarea-carbonica.

The Mercurius patient suffers from dullness of hearing on blowing the nose. Greenish, fœtid pus is discharged from the nose, which is similar to Pulsatilla, except that the color under the latter remedy is yellowish-green.

Mercurius has profuse fluent coryza of a very virulent type and the discharge being corrosive. This is somewhat similar to Arsenicum. The difference being that the coryza under Mercurius is more virulent and the discharge is acrid. Another difference is that the Mercurius patient is bathed in perspiration which does not relieve, while the Arsenic patient has a cold skin with dryness suggesting parchment.

The Mercury patient has dull, lustreless eyes, while the Belladonna patient is just the opposite, having bright lustrous eyes.

The Mercury patient also has inflammatory swelling of the lower jaw and almost complete immobility of the jaw. This is similar to Lachesis.

There is caries of the jaw under Mercurius similar to Phosphorus. This would naturally be expected when we recollect how Mercury in massive doses loosens the teeth and softens the gums in cases of salivation.

Mercurius is valuable in the sore mouth of children, with or without parasitic growths. Nitric-acid is also valuable in the same condition. The most striking characteristic of Mercurius is the indentation of the edges of the tongue by the teeth. Arsenicum-album, Iodine, Rhus-toxicodendron, and Stramonium have this same symptom.

Dr. Lippe, however, did not mention all the remedies that have this symptom. From his note-book the editor copies the following:

Tongue indented upon the edges. Arsenicum-metallicum, Arsenicum-album, Antimonium-tartaricum (Tartar-emetic), Glonoine, Hydrastis, Ignatia, Iodine, Kali-bichromicum, Kali-iodatum, Mercurius, Podophyllum, Rhus-toxicodendron, Sepia, Stramonium, Tellurium. Dr. Knerr's *Repertory to the Guiding Symptoms* gives in addition to the foregoing, Chilidonium, Syphilinum, and Vibernum. In cases of epithelioma with hypertrophy, Kali-muriaticum; in atonic dyspepsia, Sepia; in stomatitis, Hydrastis; and abuse of Mercury, Kali-iodatum.

Mercurius has swelling of the roof of the mouth in ridges as in horses.

Under Mercurius in cases of inflammation of the throat, there are stinging pains, like Ignatia. In these cases the fluid swallowed comes out through the nose. This is like Belladonna and Lachesis.

Mercurius has desire for sweet things which make his symptoms worse.

Mercurius has metallic taste in the mouth. Dr. Lippe related the case of a chemist, his patient, who could always tell when the Doctor had given him Mercurius in a potency, by the metallic taste in his mouth, after taking the pellets upon his tongue.

Mercurius has pains in the abdomen after taking cold. Nitric-acid has liability to take cold in the abdomen, causing colic. Indeed colic from taking cold in the abdomen is a keynote of Nitric-acid.

From the foregoing it will be seen that there is a continual correspondence between Mercurius and Nitric-acid. One supplements the other. One antidotes the other. Take a case coming from the old school, where the patient has been dosed to excess with either Calomel or Blue Mass, Nitric-acid is likely to be the remedy, and will do wonders in relieving the patient.

In conclusion, attention may be called to the sensation of emptiness in the abdomen, which is Dr. Guernsey's keynote for Phosphorus. This symptom can also be found under Mercurius.

---

DR. MARTIN DESCHERE.—The editor owes Dr. Deschere an apology for the astonishing transformation of his name as printed in the January number of THE HOMŒOPATHIC PHYSICIAN at page 40.

In giving the list of officers of "The Hahnemann Association of New York," the name of the President is printed "Naesteri Desebere, M. D." This absolutely unique perversion of the name of so distinguished a man as Dr. Deschere is not, as it would seem to be, a printer's blunder. It occurs exactly so in the original manuscript sent to this office for publication.

The editor noticed it in reading the proof, and at once referred to the copy, only to find it there. It was his intention to write to New York and find out what the name really was. Other duties pressing upon his attention, this resolution was forgotten, and so this strange combination of letters got into print. It is only within a few days that the attention of the editor was called to the queer thing by the Corresponding Secretary, Dr. H. D. Schenck. Hence the tardiness of this correction.

